# Platelet-Rich Plasma Modulates Neuroinflammation in an iPSC Model of Sensory Neurons and Microglia

**DOI:** 10.3390/ijms27167127

**Published:** 2026-08-08

**Authors:** Jon Mercader-Ruiz, Daniel Marijuan-Pinel, Diego Delgado, Deiene Lasuen Aguirre, Xabier Sansinanea, Jorge Guadilla, Mikel Sánchez

**Affiliations:** 1 Advanced Biological Therapy Unit, MiKS Hospital, 01010 Vitoria-Gasteiz, Spain; jon.mercader@hospitalmiks.com (J.M.-R.); daniel.marijuan@hospitalmiks.com (D.M.-P.); diego.delgado@hospitalmiks.com (D.D.); 2 Arthroscopic Surgery Unit, MiKS Hospital, 01010 Vitoria-Gasteiz, Spain; deilasuen@hotmail.com (D.L.A.); xabi.sansinanea@hospitalmiks.com (X.S.); jorge.guadilla@hospitalmiks.com (J.G.)

**Keywords:** platelet-rich plasma, neuroinflammation, neuropathic pain, microglia, sensory neurons, iPSC model

## Abstract

Neuropathic pain (NP) is driven by neuroimmune interactions where in activated microglia release pro-inflammatory mediators that sustain central sensitization. Platelet-rich plasma (PRP) is a promising therapy, but its efficacy depends on its biochemical composition. This study aimed to evaluate how modifying the molecular profile of PRP influences its capacity to modulate neuroinflammation in a human-derived co-culture model. We compared standard PRP (sPRP) and balanced protein-concentrate plasma (BPCP), enriched in extraplatelet molecules, using an iPSC direct co-culture of sensory neurons (hSNs) and microglia (hMG). Neuroinflammation was induced for 24 h with serum-free (SF) or 10% sPRP or BPCP supplementation. Neuroinflammation, microglial activation, apoptosis, and neuronal plasticity were analyzed via RT-qPCR and Luminex. Both formulations attenuated pro-inflammatory mediators. However, BPCP demonstrated superior suppressive efficacy, reducing the levels of major cytokines (IL-1β, TNF-α, IL-6, IL-8) by half as compared to sPRP. BPCP significantly decreased IL-6, IL-8, and MCP-1 protein levels; kept microglial activation markers (CD86, CTSS) downregulated; and downregulated the apoptotic cascade (BAX, CASP9, CASP3). Conversely, sPRP displayed a distinct pro-resolving and neuroprotective trend, upregulating IL-10 and TGF-β1 and rescuing the expression of *SLC12A5* (KCC2), which regulates neuronal excitability. sPRP and BPCP modulated neuroinflammation via complementary mechanisms. BPCP acted as an immunomodulatory shield suppressing macro-inflammation, while sPRP drove inflammation resolution and neuroplastic rescue.

## 1. Introduction

Neuropathic pain (NP) is a maladaptive, chronic condition caused by somatosensory nervous system lesions [1,2,3,4]. Peripheral nerve damage triggers abnormal excitability and long-lasting peripheral and central sensitization, resulting in allodynia and hyperalgesia [3,5,6]. Because current treatments are often insufficient, there is a critical need for new mechanism-based therapeutic strategies [7,8].

Evidence demonstrates that NP is not exclusively neuronal but is critically driven by neuroimmune interactions, particularly involving microglia [9,10,11]. Following nerve injury, microglia become activated and release pro-inflammatory mediators, cytokines, and neurotrophic factors that alter synaptic transmission and neuronal excitability [12,13,14,15,16]. Sustained microglial activation contributes to chronic neuroinflammation and the maintenance of pain states, making microglia a key therapeutic target in NP [17,18].

Platelet-rich plasma (PRP) is an autologous biological product enriched with platelets and a wide array of bioactive molecules capable of modulating inflammation, angiogenesis, and tissue repair [19,20,21]. PRP has recently gained attention as a potential treatment for NP [22,23,24], eliciting immune responses and neural regeneration through complex molecular interactions [25,26,27]. Moreover, emerging evidence suggests that PRP may modulate microglial activation and restore neuroimmune homeostasis [28,29]. PRP-derived mediators can reduce pro-inflammatory signaling, promote neuroprotective phenotypes in glial cells, and support axonal regeneration and remyelination [30,31]. Through these mechanisms, PRP may disrupt the inflammatory loop that sustains central sensitization and chronic pain.

Importantly, besides platelet-derived factors, the therapeutic potential of PRP is attributed to extraplatelet molecules present within its plasma fraction [32,33]. These include cytokines, extracellular vesicles, and emerging mediators such as insulin-like growth factor-1 (IGF-1) and hepatocyte growth factor (HGF), which may play roles in cell proliferation and regulating inflammation, respectively [34,35]. Recognition of PRP as a complex biological system underscores the importance of characterizing its full molecular composition to optimize its application as a targeted, mechanism-driven therapy for neuropathic pain.

Based on these considerations, the hypothesis of this work is that modifying the molecular profile of PRP by selectively enriching the extraplatelet molecules could enhance its protective capacity, allowing for more efficient regulation of neuroinflammation and cell survival in the context of neuropathic pain. Therefore, the objective of this study was to evaluate and compare the effects of standard PRP (sPRP) and balanced protein-concentrate plasma (BPCP), enriched in extraplatelet components, on neuroinflammatory modulation, microglial activation, apoptosis, and neuronal plasticity, utilizing a humanized direct co-culture model of iPSC-derived sensory neurons and microglia.

## 2. Results

### 2.1. Platelet Level Measurement

Platelet levels were measured in a plasma column (PC), sPRP, and BPCP to compare their biochemical compositions. Platelet analysis showed that the platelet contents in sPRP (452 × 10^3^ platelets/µL ± 63) and BPCP (468 × 10^3^ platelets/µL ± 61) were twice the blood basal levels measured in the PC sample (236 × 10^3^ platelets/µL ± 42), and these differences were statistically significant (** *p* = 0.001 and *** *p* = 0.0006, respectively). Moreover, there was no significant difference in platelet concentration between the sPRP and BPCP (*p* = 0.915). Meanwhile, residual leukocytes and erythrocytes were below the detection limit. According to the UCS (Universal Coding System) for PRP studies described by Kon et al. [36], the products used in this study were 24-00-11 (Table 1).

### 2.2. IFN-γ- and LPS-Induced Inflammation Was Reduced by PRP

**Figure 1:**
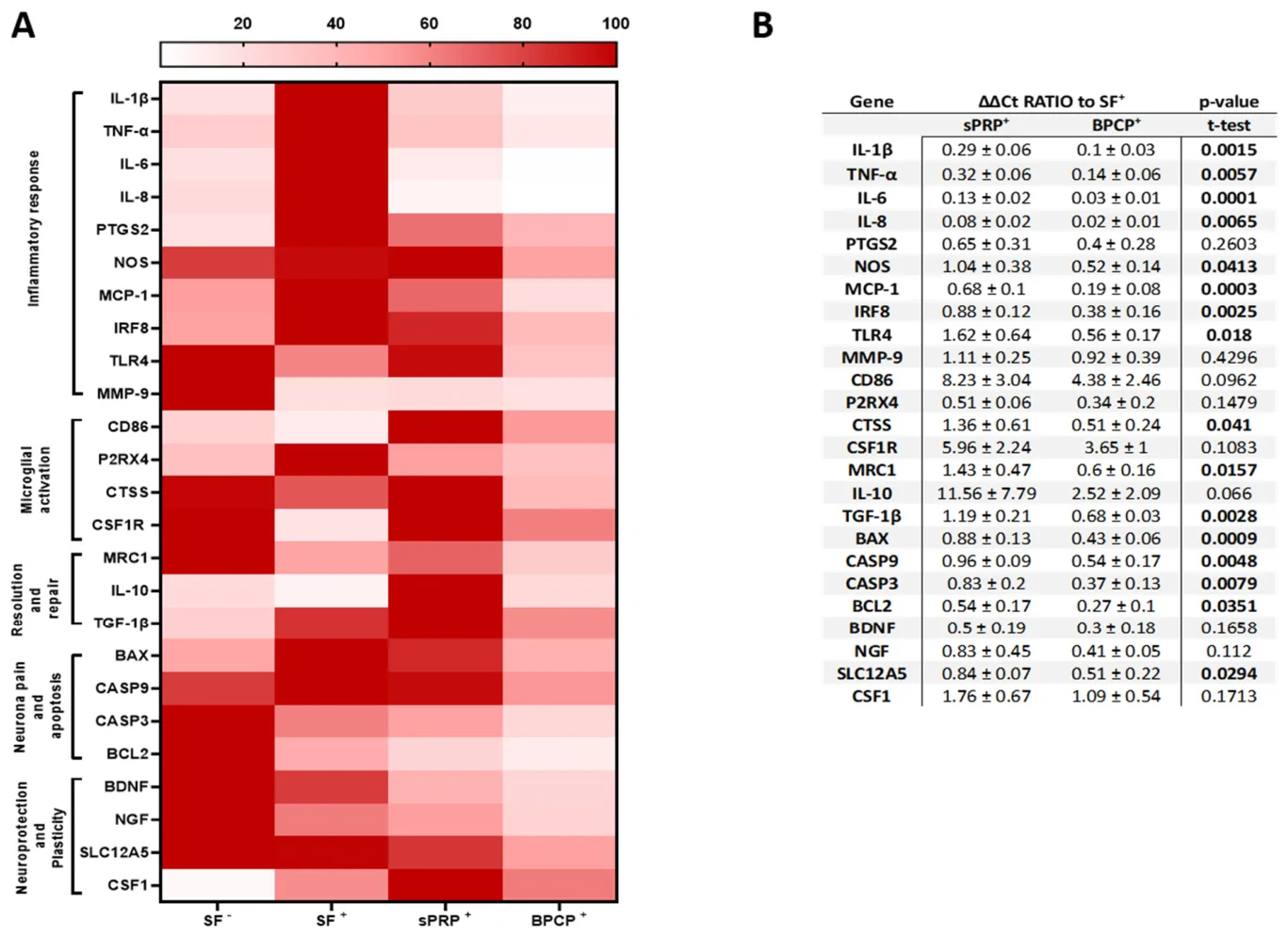
Gene expression analysis in hSNs and hMG under inflammatory conditions following PRP treatments. Relative gene expression levels were determined via RT-qPCR in hSN and hMG cells after 24 h of co-culture in the presence of LPS (50 ng/mL) and IFN-γ (10 ng/mL). The cells were subject to serum-free (SF), sPRP, or BPCP treatment. Gene expression was normalized to that of housekeeping genes and calculated using the ΔΔCt method. (**A**) A heat map of analyzed genes, including genes relating to inflammatory mediators, microglial activation, resolution and repair, neuronal pain and apoptosis, and neuroprotection and neuroplasticity. (**B**) Relative expression ratios of genes in sPRP^+^ and BPCP^+^ relative to SF^+^. Data are presented as the mean ± SD of ΔΔCt-derived fold-change relative to SF^+^. Statistical differences between sPRP and BPCP were assessed using an unpaired two-tailed Student’s *t*-test. *p*-values are indicated in the right-hand column, and significant values (*p* < 0.05) are shown in bold. “+”: inflammatory condition; “−”: non-inflammatory condition.

To characterize the molecular mechanisms underlying the effects of sPRP and BPCP treatments under inflammatory conditions, we analyzed the transcriptional profiles of 21 genes categorized into five distinct functional clusters using RT-qPCR (Figure 1A). Gene expression was analyzed from the total RNA extracted from direct co-cultures of hSNs and hMG; therefore, expression levels reflect the integrated transcriptional response of the system.

The application of IFN-γ and LPS successfully established an in vitro inflammatory model. The untreated inflamed control group (SF^+^) exhibited profound upregulation of classical pro-inflammatory cytokines and enzymes, including IL-1β, TNF-α, IL-6, IL-8, and PTGS2 (COX-2), compared to the non-inflamed control group (SF^−^). Conversely, *TLR4* and *MMP-9* transcripts showed high expression under basal conditions but were downregulated following the inflammatory challenge. Treatment with either sPRP or BPCP effectively downregulated these core pro-inflammatory mediators, significantly attenuating the inflammatory response. However, a divergence between the two treatments was observed: sPRP maintained or further elevated the expression of downstream signaling and regulatory components such as NOS, MCP-1, IRF8, and TLR4. Conversely, BPCP induced more generalized and uniform suppression across the entire set of inflammatory markers. Moreover, a comparative ratio analysis relative to the inflamed control revealed significant differences between the two PRP formulations in terms of most inflammatory markers: IL-1β (*p* = 0.0015), TNF-α (*p* = 0.0057), IL-6 (*p* = 0.0001), and IL-8 (*p* = 0.0065). BPCP reduced their relative expression by at least half in almost all cases, as compared to sPRP (Figure 1B).

The inflammatory challenge in the SF^+^ group triggered a selective increase in the receptor P2RX4, while other activation markers remained unchanged or decreased. When treated with sPRP, the microglial population underwent a distinct phenotypic shift, characterized by upregulation of CD86 and the lysosomal protease cathepsin S (*CTSS*), alongside high levels of colony-stimulating factor 1 receptor (*CSF1R*). In contrast, the BPCP formulation kept these specific microglial activation markers at low to moderate levels. In fact, BPCP significantly reduced the levels of *CTSS* as compared with sPRP (*p* = 0.041) (Figure 1B).

Moreover, the sPRP group exhibited upregulation of the anti-inflammatory cytokine IL-10 and the regulatory factor TGF-1β. This specific pro-resolving trend was remarkably stronger in the sPRP group than the BPCP group (Figure 1B).

Exposure to inflammatory stimuli activated the intrinsic apoptotic pathway, as evidenced by increased levels of the pro-apoptotic markers BAX and CASP9, coupled with simultaneous downregulation of the anti-apoptotic BCL2. While sPRP sustained elevated levels of BAX and CASP9, it prevented full execution of the cascade by limiting expression of the downstream of CASP3, as compared to the SF^+^ control. When the ratios were compared relative to SF^+^, all markers in the BPCP group showed significant differences from their counterparts in the sPRP group (Figure 1B).

The physiological expression of neurotrophic factors (BDNF, NGF) observed in the healthy baseline (SF^−^) was decreased following inflammation. Notably, sPRP demonstrated superior neuroprotective potential by strongly upregulating CSF1 and partially rescuing *SLC12A5* expression. While BPCP also showed a mild recovery effect on neuroplasticity markers, the response was substantially lower in magnitude than that induced by the sPRP treatment; the difference was statistically significant for *SLC12A5* (*p* = 0.0294) (Figure 1B).

### 2.3. BPCP Reduced Pro-Inflammatory Marker Levels as Compared to sPRP

**Figure 2:**
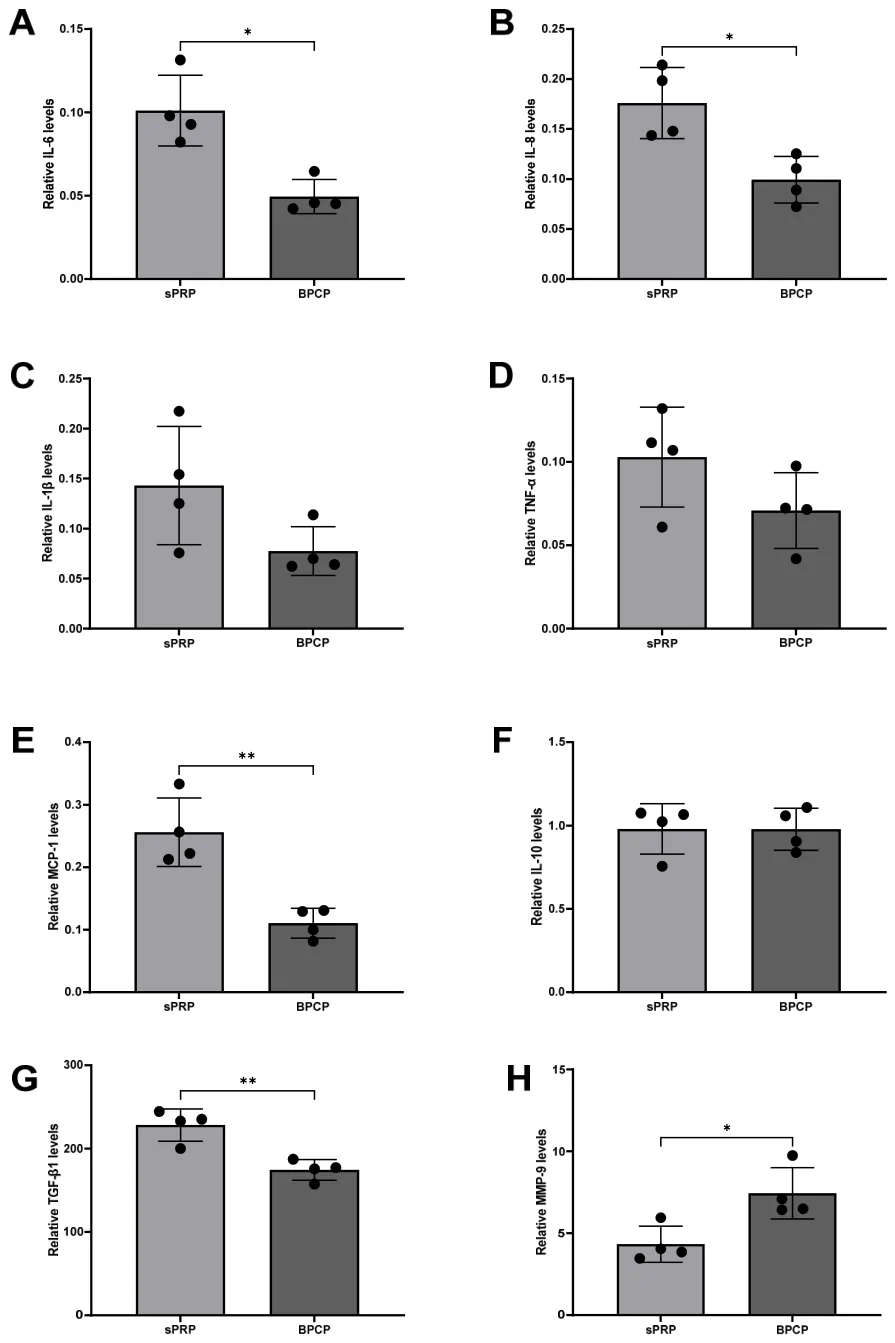
Relative protein levels in hSN and hMG co-culture under inflamed conditions. (**A**) IL-6, (**B**) IL-8, (**C**) IL-1β, (**D**) TNF-α, (**E**) MCP-1, (**F**) IL-10, (**G**) TGF-β1, and (**H**) MMP-9 levels were analyzed in cell supernatants via Luminex. Data are expressed as fold-changes relative to the serum-free (SF) group. Error bars = standard deviation (n = 4). Statistically significant differences were calculated using *t*-tests (* *p* < 0.05; ** *p* < 0.01).

Different pro-inflammatory markers were analyzed in cell culture supernatants via Luminex (Figure 2). LPS and IFN-γ effectively triggered an inflammatory response, as evidenced by significant increases in IL-1β, IL-6, IL-8, TNF-α, and MCP-1 levels in the SF^+^ control group relative to the unstimulated SF^−^ baseline (Supplementary Material, Figure S1A–D). For, IL-1β, IL-6, IL-8, and TNF-α, there were no significant differences among the three experimental groups under non-inflammatory conditions (Supplementary Material, Table S3). Nevertheless, the sPRP and BPCP groups showed a significant decrease in pro-inflammatory markers under LPS and IFN-γ stimulation compared to the SF condition (Supplementary Material, Table S2). MCP-1 levels were significantly reduced under both conditions (Supplementary Material, Tables S2 and S3).

To evaluate the effect of both PRPs, the data were normalized as fold-changes relative to SF. Under inflammatory conditions, BPCP induced significant fold-reductions in IL-6 (*p* = 0.0286), IL-8 (*p* = 0.0113), and MCP-1 (*p* = 0.0028) levels as compared to sPRP. Moreover, although the differences did not reach statistical significance, a clear trend towards a decrease in TNF-α (*p* = 0.1397) and IL-1β (*p* = 0.0571) levels was observed following BPCP as compared to sPRP (Figure 2A–D).

IL-10 and TGF-β1 were analyzed as anti-inflammatory markers. While no differences were observed simultaneously in both between the treatments under the inflamed and non-inflamed states, the BPCP treatment induced highly significant levels of IL-10 under non-inflamed conditions compared to the inflamed state (*p* = 0.0056) (Supplementary Material, Figure S1). Moreover, IL-10 levels were significantly increased in the BPCP group as compared to SF^−^ (*p* = 0.0043) and sPRP (*p* = 0.0413) (Supplementary Material, Table S3). Under inflammatory conditions, no significant differences were observed between the two PRPs (*p* > 0.999) (Figure 2E). Under both non-inflamed and inflamed conditions, both PRP formulations induced a significant increase in TGF-β1 levels as compared to the SF^+^ control (Supplementary Material, Figure S1F). Furthermore, this upregulation was significantly more pronounced in the sPRP group than in the BPCP group (*p* = 0.0034) (Figure 2G).

Finally, MMP-9 levels were measured. No significant differences were observed between the non-inflamed and inflamed conditions within each group (Supplementary Material, Figure S1). Nevertheless, under both conditions, a significant increase in the MMP-9 level was observed for both sPRP and BPCP compared to SF^+^ (Supplementary Material, Tables S2 and S3). Furthermore, a significant difference was found between the two treatments, with BPCP inducing a two-fold increase compared to sPRP (*p* = 0.0175) (Figure 2H).

## 3. Discussion

The main findings of this study are that both sPRP and BPCP significantly attenuated neuroinflammation in a direct co-culture model of hSNs and hMG in vitro *by modulating different molecular pathways*. On the one hand, BPCP suppressed inflammation and downregulated the apoptotic cascade. On the other hand, sPRP actively drove inflammation resolution and neuroplastic rescue by upregulating anti-inflammatory markers.

The establishment of an in vitro inflammatory model combining iPSC-derived hSNs and hMG provides a physiologically relevant platform to study neuroimmune interaction, which is a critical driver in the pathogenesis of NP [12,15,37]. Upon nerve injury, activated microglia disrupt neuroimmune homeostasis by releasing a cascade of pro-inflammatory cytokines and neurotoxic factors that enhance peripheral and central sensitization [38,39]. In this study, the inflammatory environment induced by IFN-γ and LPS successfully triggered the upregulation of classical pro-inflammatory mediators at both the transcriptional and translational levels, mimicking the characteristic hyperactive state seen in neurodegenerative disorders [40,41]. Crucially, the administration of both sPRP and BPCP effectively downregulated these inflammatory markers, highlighting the overall therapeutic potential of PRP products to modulate neuroinflammation [21,26,42].

However, BPCP exhibited a significantly more uniform and suppressive effect across the inflammatory markers. This capacity could be driven by its enrichment with extraplatelet molecules [32], as both formulations had the same platelet levels. At the transcriptional level, BPCP reduced the relative expression of major cytokines (*IL-1β*, *TNF-α*, *IL-6*, and *IL-8*) by at least half as compared to sPRP. This anti-inflammatory capacity was further validated through multiplex supernatant analysis, where BPCP induced significantly greater fold-reductions in IL-6, IL-8, and MCP-1 proteins under inflammatory conditions. In fact, several studies have shown that IGF-1 and HGF, which are concentrated in BPCP [32,33], can reduce inflammatory markers in vitro [43,44,45,46]. Mechanistically, these results underscore that while platelet-derived growth factors play a vital role in tissue repair [22,47], the extraplatelet plasma fraction contains crucial immunomodulatory proteins capable of synergistically reducing neuroinflammation [32].

The characterization of microglial activation phenotypes further highlighted these regulatory patterns. Microglial activation is categorized into pro-inflammatory (M1-like) and pro-resolving/neuroprotective (M2-like) states [48,49]. Moreover, it is well reported that glial cells influence neuronal function [50,51]. While it has been reported that PRP promotes the M2 phenotype [52,53], in the hSN and hMG co-culture system, sPRP treatment showed an M1-like phenotype characterized by the upregulation of *CD86* and cathepsin S (*CTSS*), alongside elevated *CSF1R* levels. Nevertheless, sPRP simultaneously upregulated the regulatory transcripts *IL-10*, *MRC-1*, and *TGF-1β*, showing a clear trend towards an anti-inflammatory environment [54]. Conversely, BPCP kept *CD86* and *CTSS* transcripts at low to moderate levels. Curiously, protein analysis revealed that the BPCP treatment pre-induced highly significant baseline levels of IL-10 under non-inflamed conditions. This suggests that BPCP might exert a protective immunomodulatory effect preventing microglia from causing excessive damage [55,56].

Furthermore, the regulation of MMP-9 highlights another vital molecular pathway. While MMP-9 transcripts were downregulated after the initial inflammatory stimulus, protein levels in the supernatant significantly increased following both PRP treatments, with two-fold induction under BPCP treatment as compared to sPRP. This mRNA–protein discrepancy is common for matrix metalloproteinases; MMP-9 is often stored intracellularly or rapidly released via exocytosis upon plasma stimulation without requiring immediate transcription at 24 h [57]. In the context of the nervous system, MMP-9 has dual functions. On the one hand, it is acutely linked to early-phase pain hypersensitivity, in which increased MMP-9 activity has been strongly associated with blood–nerve barrier disruption, neuroinflammation, and the development of NP through the activation of pro-inflammatory signaling pathways [58]. On the other hand, its sustained and controlled activity is indispensable for extracellular matrix remodeling and for helping axonal regeneration, Schwann cell migration, and remyelination [59]. Therefore, the increased MMP-9 levels observed after PRP treatment should be interpreted not as exclusively beneficial or detrimental but, rather, as part of a complex biological response for which the functional consequences are likely to depend on the timing, magnitude, and regulation of its activity.

Lastly, the impact of both PRP formulations on cell survival and neuroplasticity was analyzed. It is known that neuroinflammation activates apoptotic pathways in different cellular systems [60,61,62]. This was observed in the SF^+^ group as the upregulation of BAX and CASP9 and downregulation of the anti-apoptotic BCL2. While sPRP sustained high BAX and CASP9 and reduced CASP3, BPCP demonstrated systemic downregulation of all apoptotic transcripts (BAX, CASP9, CASP3, BCL2) relative to sPRP. This indicated that BPCP, thanks to its biochemical composition, would prevent upstream activation of the apoptotic cascade altogether by rapidly neutralizing the cytotoxic environment. On the other hand, sPRP demonstrated a possibly superior neuroprotective profile regarding neuroplasticity, as shown by its capacity to strongly upregulate *CSF1* and rescue *SLC12A5* expression as compared to BPCP. *SLC12A5* encodes K^+^/Cl^−^ cotransporter 2 (KCC2), the downregulation of which in sensory neurons is a characteristic mechanism causing central sensitization and neuropathic pain [63]. By preserving *SLC12A5* expression, sPRP displayed the possibility of a powerful mechanistic capacity to directly regulate neuronal excitability and pain signaling [64].

While this study provides valuable mechanistic insights into the differential effects of sPRP and BPCP, several limitations must be acknowledged. Firstly, the use of an in vitro co-culture system, although highly standardized and human-derived, cannot fully replicate the complex three-dimensional microenvironment and systemic immune responses of the central or peripheral nervous system in vivo. Furthermore, utilizing cells derived from a single donor may limit the generalization of the findings across broader genetic backgrounds. Secondly, due to the direct co-culture setup and the bulk extraction methodologies employed for RT-qPCR, cell-type-specific transcriptional attribution could not be definitively isolated for each marker. Future studies utilizing single-cell RNA sequencing (scRNA-seq) or fluorescence-activated cell sorting (FACS) are warranted to precisely and independently dissect the isolated transcriptional shifts within the sensory neuron and microglial populations. Thirdly, the sample size in the functional cell assays was relatively low (n = 4), primarily due to the high technical complexity and economic costs associated with maintaining and analyzing human iPSC-derived co-cultures. Finally, further proteomic profiling of the extraplatelet molecular pool would be convenient to identify the specific extraplatelet proteins primarily responsible for the superior upstream immunomodulatory shield observed in the BPCP matrix.

In summary, both PRP formulations act as powerful tools against neuroinflammation through different molecular pathways. sPRP acts as an anti-inflammatory formulation, whereas BPCP could act as a possible neuroprotective treatment by suppressing inflammation and apoptosis. These findings emphasize that the biochemical composition of PRP proteins might be crucial for the neuroprotection effect and reinforce the importance of PRP therapy customization for the treatment of chronic NP [65,66].

## 4. Materials and Methods

### 4.1. Sample Collection and PRP Preparation

#### 4.1.1. Donors

Thirty-two healthy volunteers aged between 21 and 65 years were selected for this study. Blood was collected in 9 mL samples containing 3.8% (*w*/*v*) sodium citrate and centrifuged to obtain plasma. Within each experimental group, plasma samples were combined to generate four independent pooled preparations (n = 8 donors per group). They underwent analytical tests to rule out hepatitis B, hepatitis C, HIV I/II, and HTLV I/II, in accordance with applicable regulations. This research was carried out following the principles outlined in the Declaration of Helsinki and was approved by the Institutional Ethics Committee of OSI Araba (approval number 2024-023, dated 30 May 2024). Written informed consent was obtained from all participants prior to their involvement in this study.

#### 4.1.2. Balanced Protein-Concentrate Plasma Preparation

As described by Mercader et al. [34], BPCP was obtained by centrifuging 9 mL of whole blood at 1200× *g* for 8 min at room temperature (RT) and collecting the entire PC. PCs were combined to generate four independent pooled preparations (n = 4) consisting of 8 donors per group. Platelet levels were measured in each pool. The obtained pooled PCs contained platelets and circulating molecules at levels similar to blood basal levels; therefore, this plasma fraction was employed as a reference control, providing the baseline platelet concentration against which the experimental samples were compared.

For the preparation of BPCP, 0.125 g/mL of hydroxyethyl acrylamide (HEAA) hydrogel was added to a PC and left for 5 min to absorb the plasma’s water content. The hydrogel powder was discarded by putting the plasma in a 100 µm filtration unit on top of a 50 mL Falcon tube. The sampled was centrifuged at 500× *g* for 2 min at RT to collect the BPCP. Finally, 10% CaCl_2_ (20 µL/mL) was added to the BPCP formulation, and it was kept at 37 °C to release the platelet content. The resulting BPCP lysate was sterilized via filtration through a Minisart^®^ NML Plus 0.2 μm filter (Sartorius, Goettingen, Germany). Finally, the BPCP lysate was aliquoted, frozen, and stored at −80 °C for later analysis.

#### 4.1.3. Standard Platelet-Rich Plasma Preparation

Briefly, sPRP was obtained by using a commercially available PRP kit (BTI Biotechnology Institute, Vitoria-Gasteiz, Spain) and centrifuging 9 mL of blood at 580× *g* for 8 min at RT. A 2 mL sample of the plasma fraction present over the red blood cell fraction was collected, avoiding white blood cells from the ‘buffy coat’ layer. As mentioned above, the obtained plasma fractions were randomly assigned into four groups, each comprising samples from eight individuals. Within each group, the plasma fractions were pooled to generate four composite preparations (n = 4). Platelet levels were measured in each pool. Then, 10% CaCl_2_ (20 μL/mL) was added to the sPRP formulation to release the platelet content and initiate clot formation. The resulting sPRP lysate was sterilized via filtration through a Minisart^®^ NML Plus 0.2 μm filter (Sartorius, Goettingen, Germany). Finally, the plasma was aliquoted, frozen, and stored at −80 °C for later analysis.

### 4.2. Cell Cultures

#### 4.2.1. Human Sensory Neuron Cultures

Human induced pluripotent stem cell (iPSC)-derived sensory neurons were purchased from *bit.bio* (io1024; Cambridge, UK). The cells were derived from a healthy Caucasian adult male and induced into human sensory neurons (hSNs) by the manufacturer. Stabilization and maturation were carried out by our team according to the manufacturer’s protocol. The cells (57 × 10^3^ cells/well) were directly seeded into a 48-well plate (Costar, Corning, NY, USA) and maintained until co-culture for 7 days at 37 °C in a humid atmosphere containing 5% CO_2_.

#### 4.2.2. Human Microglia Cultures

iPSC-derived microglia were purchased from *bit.bio* (io1021; Cambridge, UK). The cells were derived from a healthy Caucasian adult male and were induced into human microglia (hMG) by the manufacturer. Stabilization and maturation were carried out by our team according to the manufacturer’s protocol. The cells (37 × 10^3^ cells/well) were directly seeded into a 48-well plate (Costar, Corning, NY, USA) and maintained until co-culture for 10 days at 37 °C in a humid atmosphere containing 5% CO_2_.

#### 4.2.3. Co-Culture of SN and MG Cells and Sample Collection

**Scheme 1:**
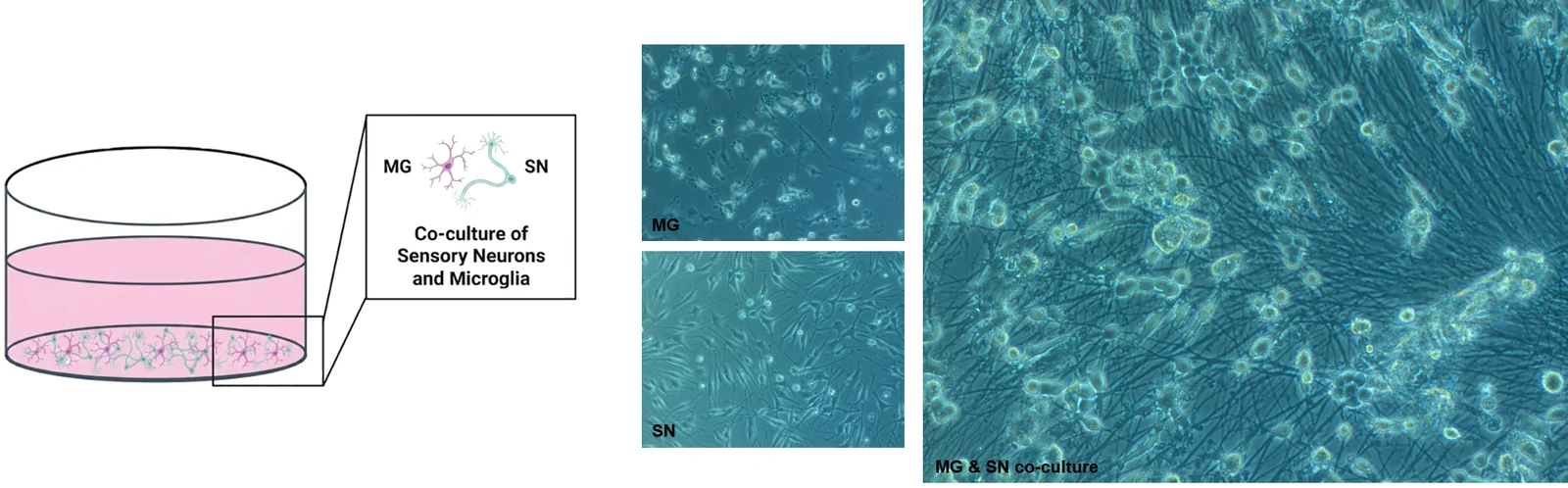
Setup and morphological characterization of a sensory neuron–microglia co-culture model. A schematic diagram illustrating the co-culture system containing sensory neurons (SNs) and microglia (MG). The inset provides a conceptual diagram of the cell-to-cell interaction. Images at 10× magnification of the SNs, MG, and co-culture system are shown.

For the hSN and hMG cell co-culture (Scheme 1), Bespoke medium (BM) was used in accordance with the manufacturer’s recommendations. After the culture medium was removed from the hSNs, hMG were gently detached in BM, and 250 µL of cell suspension containing 13.5 × 10^3^ cells was seeded on top of the hSNs. The co-cultured cells were maintained for 7 days at 37 °C in a humidified atmosphere with 5% CO_2_, and 50% of the medium was changed every 48 h.

On day 8 of co-culture, the cells were treated for 24 h with 10 ng/mL IFN-γ (285-IF-100/CF; Bio-Techne, Minneapolis, MN, USA) and 50 ng/mL LPS (L2630; Sigma-Aldrich. St. Louis, MO, USA) to induce inflammation, in addition to BM supplemented with 10% (*v*/*v*) sPRP or BPCP. A serum-free (SF) condition was used to provide a control group. Cells not treated with IFN-γ and LPS were used as a negative control. Finally, culture supernatants were collected and stored at −80 °C for analysis.

### 4.3. Gene Expression Analysis

After 24 h of treatment, gene expression analysis was carried out for both the hSN and hMG cells. The cells were lysed using 1 mL of TRIzol (15596026, Thermo Fisher Scientific, Waltham, MA, USA). Total RNA extraction, quantification, and quantitative reverse real-time PCR (RT-qPCR) from the cell cultures was performed using the Genetics, Epigenetics, and Cellular Biology platform of Bioaraba Health Research Institute (Vitoria-Gasteiz, Spain).

#### 4.3.1. Total RNA Extraction, Quantification, and Quality Control

Total RNA was extracted from cells stored in TRIzol using the Direct-zol™ RNA Miniprep kit (Zymo Research, Irvine, CA, USA), following the manufacturer’s instructions. Briefly, the cells were thawed and carefully homogenized, and RNA was purified using the silica columns included in the kit, which allow for the removal of genomic DNA and organic contaminants without the need for alcohol precipitation. In addition, during the purification process, the samples were treated with DNase I to ensure that the isolated RNA was intact and free of DNA. The RNA was eluted in nuclease-free water and stored at −80 °C until use.

RNA concentrations were determined using the Qubit™ RNA HS Assay Kit (Thermo Fisher Scientific, Waltham, MA, USA), using a Qubit™ 3.0 fluorometer. RNA purity and quality were further assessed by means of spectrophotometry with a NanoDrop™ One C (Thermo Fisher Scientific), considering the A260/280 and A260/230 ratios to estimate sample purity.

#### 4.3.2. Quantitative Reverse Real-Time PCR (RT-qPCR)

Gene expression was analyzed by means of two-step RT-qPCR. First, total RNA was reverse transcribed using the High-Capacity cDNA Reverse Transcription Kit with RNase Inhibitor (Applied Biosystems, Foster City, CA, USA; Thermo Fisher Scientific, Waltham, MA, USA), following the manufacturer’s recommendations. Up to 1 µg of total RNA was used as a template in 20 µL reactions under the following conditions: 25 °C for 10 min, 37 °C for 2 h, and 85 °C for 5 min for enzyme inactivation. The cDNA obtained was immediately used in quantitative amplification.

qPCR was performed with TaqMan™ Universal PCR Master Mix (Applied Biosystems) in a 7500 Real-Time PCR System (Applied Biosystems, Thermo Fisher Scientific), using custom-designed plates (Custom TaqMan™ Array Plates, Thermo Fisher Scientific) that included specific probes for the genes of interest (Supplementary Information, Table S1) as well as negative controls (18sRNA) to verify the absence of contamination. Each qPCR reaction had a final volume of 20 µL.

The amplification program consisted of initial denaturation at 95 °C for 10 min, followed by 40 cycles of 95 °C for 15 s and 60 °C for 1 min. Ct values were obtained automatically using Applied Biosystems 7500 v2.0.3 software, and relative expression was calculated via the ΔΔCt method, using housekeeping genes for normalization.

### 4.4. Biochemical Analysis

#### 4.4.1. Platelet Level Measurement

Platelet levels were measured in PC, sPRP, and BPCP using a Mindray BC-20s hematology analyzer (Mindray, Shenzhen, China).

#### 4.4.2. Analysis of Secreted Proteins

The levels of several cytokines and proteins were detected in co-culture supernatant by means of immunology multiplex assays using different commercially available MILLIPLEX^®^ panels. IL-1β, IL-6, IL-8, IL-10, MCP-1, MMP-9, and TNF-α were analyzed using HPLX1-115SP-12M PLEXpedition Screening panel MIXMATCH (Merck, Darmstadt, Germany). TGF-β1 levels were measured using a TGFBMAG-64K-01 Single Plex MAGNETIC Bead kit (Merck, Darmstadt, Germany). Plates were analyzed using a Luminex xMAP analyzer (Thermo Fisher Scientific, Waltham, MA, USA). Technical duplicates of all samples were carried out. All protein levels were measured via absorbance, and the corresponding concentrations were calculated by means of calibration curves (Four Parametric Logistic Regression, 4PL).

### 4.5. Statistical Analysis

Data distributions were assessed using Shapiro–Wilk’s normality test. Different variables are presented as mean ± SD values for parametric data. The statistical significance of differences between two groups was determined with Student’s *t*-test. Differences among more than two groups were validated with one-way or two-way ANOVA followed by Tukey/Dunnett’s multiple comparison test. For non-parametric data, the Kruskal–Wallis test was performed. Differences were considered statistically significant when *p* < 0.05. GraphPad Prism^®^ version 9.5 (GraphPad Software, San Diego, CA, USA) was used for the statistical analyses.

## 5. Conclusions

This study demonstrated the protective capacity of different PRP formulations, establishing them as a viable alternative treatment for NP that acts by directly targeting and modulating the neuroinflammatory response. Both sPRP and BPCP were observed to be highly effective tools capable of modulating neuroinflammation in an in vitro hSN and hMG co-culture model. Crucially, our findings revealed that these formulations operate via distinct and complementary biological mechanisms, highlighting the importance of the biochemical composition of PRP. Taken together, these insights demonstrate that modifying PRP’s composition could allow for personalized plasma therapies, providing better clinical tools to treat chronic NP.

## Figures and Tables

**Table 1:** Summary of characteristics for PRP pool.

	sPRP	BPCP
**PRP Preparation**		
Initial blood volume	9 mL per tube	9 mL per tube
Anticoagulant	Sodium citrate 3.8% (wt/V)	Sodium citrate 3.8% (wt/V)
System	Closed	Open
Centrifugation	Yes	Yes
Number	1	1
Speed	580 g—8 min	1200 g—8 min
Water absorption	No	Yes
Method		HEAA hydrogel
Hydrogel concentration		0.125 g mL^−1^
Contact time		5 min
Final PRP volume	2 mL per tube	2 mL per tube *
2. **PRP Characteristics**		
PRP type	24-00-11^20^	24-00-11^20^
Platelets	452 × 10^3^/µL ± 63.38	468 × 10^3^/µL ± 60.65
Red blood cells	<0.01 × 10^6^/µL	<0.01 × 10^6^/µL
White blood cells	<0.05 × 10^6^/µL	<0.05 × 10^6^/µL
Neutrophils	---	---
Lymphocytes	---	---
Monocytes	---	---
Eosinophils	---	---
Basophils	---	---
Activation	CaCl_2_ (10% wt/vol)	CaCl_2_ (10% wt/vol)
3. **Application Characteristics**		
Dose	10%	10%
Direct/Indirect	Direct	Direct
4. **Other Remarkable PRP and Study Features**		
The product added to the cell cultures was the platelet lysate obtained following the activation of PRP with CaCl_2_ (10%). sPRP and BPCP had double the basal blood level of platelets.		

sPRP: standard platelet-rich plasma; BPCP: balanced protein-concentrate plasma; HEAA: hydroxyethyl acrylamide. * The final volume of BPCP depended on the amount of plasma column processed with the hydrogel. For a detailed description of the preparation process, please refer to Section 4.1.2 of the Materials and Methods section.

## Data Availability

The data presented in this study are available on request from the corresponding author.

## Supplementary Materials

The following supporting information can be downloaded at https://www.mdpi.com/article/10.3390/ijms27167127/s1.

## Abbreviations

The following abbreviations are used in this manuscript:

BDNF	Brain-derived neurotrophic factor
BM	Bespoke medium
BPCP	Balanced protein-concentrate plasma
FACS	Fluorescence-activated cell sorting
HEAA	Hydroxyethyl acrylamide
HGF	Hepatocyte growth factor
hMG	Human microglia
hSN	Human sensory neuron
IFN-γ	Interferon gamma
IGF-1	Insulin-like growth factor 1
iPSC	Induced pluripotent stem cell
LPS	Lipopolysaccharide
NGF	Nerve growth factor
NP	Neuropathic pain
PRP	Platelet-rich plasma
RT	Room temperature
RT-qPCR	Quantitative reverse real-time PCR
scRNA-seq	Single-cell RNA sequencing
SF	Serum-free
sPRP	Standard platelet-rich plasma
TGF-1β	Transforming growth factor beta 1
UCS	Universal coding system
