# Supplementary material for: Platelet-Rich Plasma Modulates Neuroinflammation in an iPSC Model of Sensory Neurons and Microglia
Source: Int J Mol Sci. 2026 Aug 8;27(16):7127. doi: 10.3390/ijms27167127 (PMC13513699; doi:10.3390/ijms27167127)
Supplement: Supplementary file 1 [file ijms-27-07127-s001.zip › ijms-4440106-supplementary.pdf]

Supplementary material

Table S1: Primer sequences used for RT-qPCR. All primers were acquired from Thermofisher Scientific, as well as custom-designed plates. Asterisks indicate housekeeping genes used for data normalization. TaqMan® Assay IDs ending with "_m" indicate assays whose probe spans an exon junction and will not detect genomic DNA. "_g" indicates an assay that may detect genomic DNA; the assay primers and probe may also be located within a single exon. "_s" indicates an assay whose primers and probe are designed within a single exon and will therefore, by definition, detect genomic DNA. Additional information for each TaqMan® Assay is available from Applied Biosystems.

Gene Name	TaqMan Assay ID	Transcript
**18SRNA***	Hs99999901_s1	X03205.1 (mRNA)
**ARG-1**	Hs00163660_m1	NM_000045.3
**BAX**	Hs00180269_m1	NM_001291428.1
**BCL2**	Hs04986394_s1	NM_000633.2
**BDNF**	Hs02718934_s1	NM_001709.4
**CASP3**	Hs00234387_m1	NM_004346.3
**CASP9**	Hs00962278_m1	NM_001229.4
**CTSS**	Hs01080392_m1	NM_004079.4
**CCR2**	Hs01560352_m1	NM_001123041.2
**MRC1**	Hs07288635_g1	NM_002438.3
**CD86**	Hs01567026_m1	NM_175862.4
**PTGS2**	Hs00153133_m1	NM_000963.3
**CSF1**	Hs00174164_m1	NM_000757.5
**CSF1R**	Hs00911250_m1	NM_001288705.1
**CX3CL1**	Hs00171086_m1	NM_002996.4
**CX3CR1**	Hs01922583_s1	NM_001171174.1
**IL10**	Hs00961622_m1	NM_000572.2
**IL-13**	Hs00174379_m1	NM_002188.2
**IL-1β**	Hs01555410_m1	NM_000576.2
**IL-4**	Hs00174122_m1	NM_000589.3
**IL-6**	Hs00174131_m1	NM_000600.4
**CXCL8**	Hs00174103_m1	NM_000584.3
**NOS2**	Hs01075529_m1	NM_000625.4
**IRF8**	Hs00175238_m1	NM_002163.2
**SLC12A5**	Hs00221168_m1	NM_020708.4
**CCL2**	Hs00234140_m1	NM_002982.3
**MMP-9**	Hs00957562_m1	NM_004994.2
**NGF**	Hs00171458_m1	NM_002506.2
**P2RX4**	Hs00902156_g1	NM_002560.2
**TGF-β1**	Hs00998133_m1	NM_000660.5
**TLR4**	Hs00152939_m1	NM_138554.4
**TNF-α**	Hs00174128_m1	NM_000594.3

Table S2: Protein levels in cell culture media of hSN and hMG co-culture under inflammatory conditions induced with LPS (50 ng/mL) and IFN-γ (10 ng/mL) following treatment with sPRP and BPCP formulations. Statistically significant differences were calculated using one-way ANOVA (n = 4). Statistics marked in bold represent statistical significance between groups (A, B or C).

Gene	Protein levels (pg/mL)			p-value		
	SF (A)	sPRP (B)	BPCP (C)	A*B	A*C	B*C
**IL-6**	5506.08	556.49	272.12	**<0.0001**	**<0.0001**	0.1337
**IL-8**	7411.45	1303.98	735.84	**<0.0001**	**<0.0001**	0.5563
**IL-1β**	16.4	2.345	1.2725	**0.0004**	**0.0002**	0.7787
**TNF-α**	434.085	44.64	30.73	**<0.0001**	**<0.0001**	0.4608
**MCP-1**	5421.09	1388.17	612.313	**<0.0001**	**<0.0001**	**0.0164**
**IL-10**	1.94	1.9025	1.8975	0.9919	0.9897	0.9998
**TGF-β1**	10.63	2426.95	1855.01	**<0.0001**	**<0.0001**	**0.0037**
**MMP-9**	1030.52	4464.46	7668.92	**0.0423**	**0.0015**	**0.0236**

Table S3: Protein levels in cell culture media of hSN and hMG co-culture following treatment with sPRP and BPCP formulations under no inflammatory environment. Statistically significant differences were calculated using one-way ANOVA (n = 4). Statistics marked in bold represent statistical significance between groups (A, B or C).

Gene	Protein levels (pg/mL)			p-value		
	SF (A)	sPRP (B)	BPCP (C)	A*B	A*C	B*C
**IL-6**	52.585	54.81	37.58	0.9673	0.2847	0.1157
**IL-8**	499.385	493.313	354.88	0.9996	0.3623	0.1143
**IL-1β**	1.18	0.6075	-	ns	ns	ns
**TNF-α**	4.9	6.085	5.5675	0.7318	0.9028	0.9118
**MCP-1**	537.16	318.36	247.57	**0.001**	**0.0002**	0.0903
**IL-10**	1.125	2.34	3.62	0.1069	**0.0043**	**0.0413**
**TGF-β1**	10.25	2540.06	1606.95	**0.0002**	**0.0035**	**0.0197**
**MMP-9**	1702.09	3688.12	7382.34	**0.1**	**0.0005**	**0.0021**

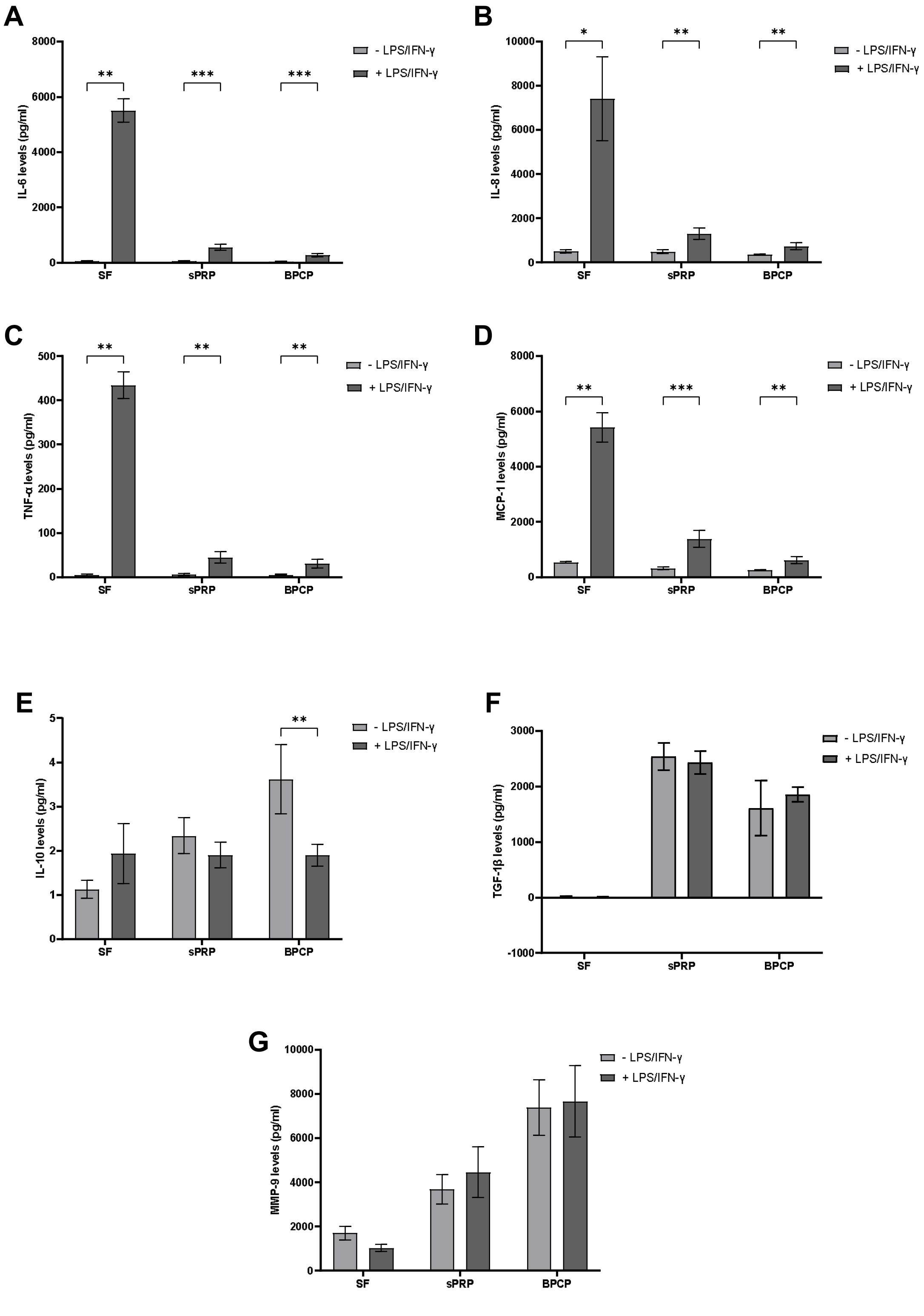

Figure S1: Protein levels under inflammatory and non-inflammatory conditions. IL-6 (A), IL-8 (B), TNF-α (C), MPC-1 (D), IL-10 (E), TGF-β (F) and MMP-9 (G) were measured in hSN and hMG co-culture supernatant by Luminex and ELISA. IL-1β was not detected under non-inflammatory conditions. Error bars = standard deviation (n = 4). Statistically significant differences were calculated using Student's t-test (* $p < 0.05$; ** $p < 0.01$; *** $p < 0.001$).
